# ICA69 regulates activity-dependent synaptic strengthening and learning and memory

**DOI:** 10.3389/fnmol.2023.1171432

**Published:** 2023-05-12

**Authors:** Shu-Ling Chiu, Chih-Ming Chen, Richard L. Huganir

**Affiliations:** ^1^Institute of Cellular and Organismic Biology and Neuroscience Program of Academia Sinica (NPAS), Academia Sinica, Taipei, Taiwan; ^2^Solomon H. Snyder Department of Neuroscience and Kavli Neuroscience Discovery Institute, Johns Hopkins University School of Medicine, Baltimore, MD, United States

**Keywords:** AMPA receptor trafficking, PICK1, synaptic plasticity, LTP, hippocampus dependent learning

## Abstract

Long-term potentiation (LTP) is one of the major cellular mechanisms for learning and memory. Activity-dependent increases in surface AMPA receptors (AMPARs) are important for enhanced synaptic efficacy during LTP. Here, we report a novel function of a secretory trafficking protein, ICA69, in AMPAR trafficking, synaptic plasticity, and animal cognition. ICA69 is first identified as a diabetes-associated protein well characterized for its function in the biogenesis of secretory vesicles and trafficking of insulin from ER, Golgi to post-Golgi in pancreatic beta cells. In the brain, ICA69 is found in the AMPAR protein complex through its interaction with PICK1, which binds directly to GluA2 or GluA3 AMPAR subunits. Here, we showed that ICA69 regulates PICK1's distribution in neurons and stability in the mouse hippocampus, which in turn can impact AMPAR function in the brain. Biochemical analysis of postsynaptic density (PSD) proteins from hippocampi of mice lacking ICA69 (*Ica1* knockout) and their wild-type littermates revealed comparable AMPAR protein levels. Electrophysiological recording and morphological analysis of CA1 pyramidal neurons from *Ica1* knockout also showed normal AMPAR-mediated currents and dendrite architecture, indicating that ICA69 does not regulate synaptic AMPAR function and neuron morphology at the basal state. However, genetic deletion of ICA69 in mice selectively impairs NMDA receptor (NMDAR)-dependent LTP but not LTD at Schaffer collateral to CA1 synapses, which correlates with behavioral deficits in tests of spatial and associative learning and memory. Together, we identified a critical and selective role of ICA69 in LTP, linking ICA69-mediated synaptic strengthening to hippocampus-dependent learning and memory.

## 1. Introduction

Long-term potentiation (LTP) has long been considered a key cellular mechanism for learning and memory (Bliss and Collingridge, 1993; Nabavi et al., 2014). Activity-dependent increase in the surface delivery of AMPA receptors (AMPARs) is crucial for the enhanced synaptic efficacy during LTP (Huganir and Nicoll, 2013; Diering and Huganir, 2018). AMPARs conduct the majority of fast excitatory synaptic transmission in the brain and are tetrameric receptors composed of subunits GluA1–4. To adjust synaptic efficacy, AMPARs are dynamically regulated through continual membrane insertion, lateral diffusion, synapse trapping and subsequent endocytosis, recycling to the cell surface, or lysosomal degradation (Anggono and Huganir, 2012). Two major intracellular trafficking pathways regulate the surface delivery of AMPARs and synaptic transmission: the de novo secretory pathway in which newly synthesized AMPARs are delivered onto the membrane and the recycling pathway in which endocytosed AMPARs can return to the membrane. While several studies have demonstrated the essential role of the recycling pathway in LTP (Park et al., 2004; Chiu et al., 2017; Moretto and Passafaro, 2018), the involvement of the secretory pathway is less explored. This study aimed to explore the contribution of AMPAR secretory trafficking in synaptic plasticity and animal behavior by studying a secretory trafficking protein, ICA69 (Islet cell autoantigen of 69 kDa).

ICA69 was first identified as an autoantigen associated with type 1 diabetes (Pietropaolo et al., 1993). However, ICA69 alone is not obligatory for type 1 diabetes because *Ica1*-knockout (KO) mice showed no obvious phenotype and aged normally unless they were crossed with non-obese diabetic mice, a well-established animal model for type 1 diabetes (Winer et al., 2002). Nevertheless, ICA69 function in pancreatic beta cells is well characterized as a Rab2 effector to regulate endoplasmic reticulum (ER) to Golgi trafficking of insulin secretory granules (Buffa et al., 2008). ICA69 also promotes the budding and post-Golgi trafficking of insulin granules in collaboration with PICK1 (protein interacting with C-kinase 1). As a result, mice lacking either ICA69 or PICK1 developed diabetes-like phenotypes, such as glucose intolerance, due to defective insulin secretion when they turned half to 1 year old (Cao et al., 2013). Consistently, PICK1 coding variants are identified in diabetic patients (Andersen et al., 2022). These results suggest a role for ICA69 and PICK1 in late-onset type 2 diabetes by regulating membrane fission in the biogenesis of insulin secretory vesicles. In the pituitary gland, ICA69 and PICK1 also work together to regulate the budding of growth hormone secretory vesicles from Golgi to ensure proper body growth in flies and mice (Holst et al., 2013). Interestingly, PICK1 is a major scaffold protein for AMPAR-mediated receptor trafficking and is therefore crucial for multiple forms of AMPAR-associated plasticity, including both the LTP and long-term depression (LTD) forms of Hebbian plasticity and homeostatic plasticity (Steinberg et al., 2006; Terashima et al., 2008; Citri et al., 2010; Volk et al., 2010; Anggono et al., 2011; Jaafari et al., 2012). Like PICK1, ICA69 also contains a BAR domain that allows ICA69-PICK1 heteromerizations or their self-homomerization. Importantly, the BAR domain and the amphipathic helix N-terminal to the BAR domains of ICA69, as well as the BAR domain of PICK1 confer their phospholipid binding and membrane curvature sensing capabilities for membrane remodeling and membrane trafficking (Jin et al., 2006; Holst et al., 2013; Mallik et al., 2017; Herlo et al., 2018). Given that ICA69 is a major binding partner of PICK1 and is part of the PICK1/AMPAR protein complex (Cao et al., 2007), ICA69 may also regulate AMPAR trafficking and affect excitatory synaptic transmission and brain function. Indeed, we have found that overexpression of ICA69 reduces synaptic targeting of AMPARs in cultured neurons (4). However, the role of ICA69 at glutamatergic synapses *in vivo* and its impact on cognitive function remain elusive. Besides AMPARs, PICK1 also interacts with F-actin and Arp2/3 to promote dendrite outgrowth in cultured neurons (Rocca et al., 2008), raising the possibility that ICA69 may regulate neuronal morphology via its interaction with PICK1.

Here, we report that ICA69 determines the subcellular distribution of PICK1 in cultured neurons and the stabilization of PICK1 in the brain. ICA69 does not regulate basal synaptic AMPAR function because AMPAR-mediated currents and major AMPAR subunits in the postsynaptic density (PSD) isolated from *Ica1*-knockout mice are normal. ICA69 also does not affect dendrite architecture. However, *Ica1-*KO mice exhibited specific impairment in NMDAR-dependent LTP, but not NMDAR- or mGluR-dependent LTD, which correlates with their behavioral deficits in hippocampal-dependent spatial and associative learning and memory. Together, our results show that ICA69 has a specific role in activity-dependent synaptic strengthening and plays a critical role in hippocampus-dependent learning and memory.

## 2. Materials and methods

### 2.1. Animals

All animal procedures were performed in accordance with the regulations of Animal Care Committees. *Ica1* mice (RRID: *MGI: 96391)* were purchased from the Jackson Laboratory and backcrossed to C57BL/6 background for more than 10 generations. Male and female *Ica1* littermates were gender-separated and group housed in two to five mice per cage. Mice were used at either juvenile (postnatal day 21–23) or adult (2–5 months, except for PSD prep in which two pairs of 7-month-old mice were included). Sprague Dawley rats (Harlan Laboratories) were used for hippocampal or cortical cultures at embryonic day 18 as described below. All animals were housed in a standard 12-h light/12-h dark cycle.

### 2.2. DNA constructs

A cDNA encoding the full-length ICA69 was subcloned into a vector downstream of CMV promoter in a pEGFP backbone (Clontech). Myc-tagged full-length, N-term, or C-term ICA69 was generated using a standard overlap extension PCR protocol. GFP and myc-tagged PICK1 were generated as previously described (Cao et al., 2007).

### 2.3. Cell cultures and transfection

HEK293T cells (ATCC) were grown in DMEM supplemented with 10% FBS, 50 U/mL penicillin, and 50 μg/mL streptomycin. Cells were transfected with 1 μg of each plasmid when co-transfected, and an empty vector was used to keep the total amount of DNA constant when only ICA69 or PICK1 was singly transfected. Lipofectamine 2000 (Invitrogen) was used for transfection following the manufacturer's instructions, and cells were processed 2 days after transfection. Hippocampal neurons from embryonic day-18 rat pups were plated onto poly-L-lysine-coated coverslips or plates in 5% horse serum (HS)-containing Neurobasal Medium with freshly added supplements (2% B27, 2 mM GlutaMAX, 50 U/ml penicillin, and 50 μg/streptomycin). Neurons were switched to serum-free Neurobasal Medium with supplements 1-day post-seeding and fed once a week with same medium and supplements. For staining, hippocampal neurons were plated at a density of 100,000 cells per well into 12-well tissue culture plates. Neurons were transfected at DIV17-18 using Lipofectamine 2000 (Invitrogen) following the manufacturer's manual, and the cells were used 1 day after.

### 2.4. Co-immunoprecipitation

HEK cells were lysed in lysis buffer (1% NP-40, 200 mM NaCl, 50 mM sodium fluoride, 5 mM sodium pyrophosphate, 20 mM HEPES (pH 7.4), and protease inhibitors). Cell lysates were collected, passed through a 26-gauge needle 12 times, and centrifuged at maximum speed in the cold room for 15 min. Approximately 250 μg protein was first incubated with 2 μl serum against PICK1 or 2 μg purified antibody against ICA69 (Cao et al., 2013) for 1 h followed by adding 10 μl Protein A or G beads at 4°C for 2 h. Beads were washed four times with lysis buffer without protease inhibitors, and immune complexes were eluted in a 2x SDS (sodium dodecyl sulfate) sample buffer with heating at 65°C for 10 min. Eluates were resolved by 8% SDS–polyacrylamide gel electrophoresis (PAGE), transferred to nitrocellulose membrane, and immunoblotted to examine the protein of interest using specific primary antibodies and corresponding secondary antibodies. The immunoreactive signals were visualized by detected simultaneously with a Li-Cor Odyssey CLx IR imaging system (Li-Cor).

### 2.5. Culture neuron imaging and image analysis

For ICA69 and PICK1 dendritic distribution assay, hippocampal neurons were transfected with GFP, HA-tagged ICA69 (either full-length, N-term, or C-term), and myc-tagged PICK1 for overnight before fixing with parafix (4% paraformaldehyde/4% sucrose in PBS) at room temperature. After washing with PBS and blocking with 3% BSA, cells were permeabilized with 0.1% Triton X-100 for 15 min and incubated with chicken anti-GFP, rabbit anti-HA and mouse anti-myc to visualize cell morphology and soma vs. dendrite localization of ICA69 and PICK1 with corresponding secondary antibodies conjugated with Alexa Fluor 488, 546, and 647. Somatic area and three dendritic segments (0–20, 20–40, and 40–60 μm away from soma) on the primary dendrite of each neuron were isolated and outlined from GFP mask in ImageJ to quantify mean fluorescent intensity in each region of interest. ICA69/PICK1 colocalization images were generated in Imaris software (Oxford Instruments).

### 2.6. PSD preparation

Hippocampus tissues from adult (2–7 months old) *Ica1* WT and KO littermates were homogenized by passage through a 26-gauge needle, 12 times, in homogenization buffer (320 mM sucrose, 5 mM sodium pyrophosphate, 1 mM EDTA, 10 mM HEPES (pH 7.4), 200 nM okadaic acid, and protease inhibitors). The homogenate was centrifuged at 800xg for 10 min at 4°C to yield post-nuclear pelleted fraction 1 (P1) and supernatant fraction 1 (S1). S1 was further centrifuged at 15,000xg for 20 min at 4°C to yield P2 and S2. P2 was resuspended in Milli-Q water, adjusted to 4 mM HEPES (pH 7.4) from a 1 M HEPES stock solution, and incubated with agitation at 4°C for 30 min. The suspended P2 was centrifuged at 25,000xg for 20 min at 4°C to yield LP1 and LS2. LP1 was resuspended in 50 mM HEPES (pH 7.4), mixed with an equal volume of 1% triton X-100, and incubated with agitation at 4°C for 15 min. The PSD was generated by centrifugation at 32,000xg for 20 min at 4°C. The final PSD pellet was resuspended in 50 mM HEPES followed by protein quantification and Western blot analysis.

### 2.7. Intracellular whole-cell recordings

Juvenile (p21-23) mice were anesthetized with the inhalation anesthetic isoflurane prior to decapitation. Brains were rapidly dissected out and placed in ice-cold, oxygenated (95% O_2_ and 5% CO_2_) low-Ca^2+^/high-Mg^2+^ dissection buffer (2.6 mM KCl, 1.25 mM NaH_2_PO_4_, 26 mM NaHCO_3_, 211 mM sucrose, 11 mM glucose, 0.5 mM CaCl_2_, and 7 mM MgCl_2_). In all, 350 μm transverse slices from dorsal hippocampus were prepared using a vibratome (Leica; VT1200s) in dissection. Slices were then transferred to a static submersion chamber filled with oxygenated ACSF1 (119 mM NaCl, 2.5 mM KCl, 1 mM NaH_2_PO_4_, 2.5 mM CaCl_2_, 1.3 mM MgSO_4_, 26.2 mM NaHCO_3_, and 11 mM glucose) at room temperature allowing recovery for at least 1 h before recording.

For spontaneous AMPA mEPSC recording, slices were perfused in ACSF1 in the presence of 1 μM TTX, 100 μM picrotoxin, and 50 μM APV at a flow rate of ~2 ml/min. Whole-cell recording pipettes (3–6 MΩ) were filled with internal solution (115 mM Cs-MeSO_3_, 0.4 mM EGTA, 5 mM TEA-Cl, 2.8 mM NaCl, 20 mM HEPES, 3 mM Mg-ATP, 0.5 mM Na_2_-GTP, pH 7.2, osmolality 295–300 mOsm). Hippocampal CA1 neurons were patched and held at −70 mV holding potential, and recording was performed at room temperature. Upon entering whole-cell mode, we allowed 5 min for dialysis of the intracellular solution before collecting data. Signals were measured with MultiClamp 700B amplifier and digitized using a Digidata 1440A analog-to-digital board. Data acquisition was performed with pClamp 10.2 software and digitized at 10 kHz. mEPSCs were detected with a template-matching algorithm in Clampfit 10.2 software. All equipment and software are from Axon Instruments/Molecular Devices. Averaged mEPSC amplitude and frequency were calculated from at least 100 events for each cell.

### 2.8. Extracellular field recordings

After slicing, a cut between CA3 and CA1 of each hippocampal slice was made to minimize recurrent activity during recording. Slices were then transferred to a static submersion chamber filled with oxygenated ACSF2 (125 mM NaCl, 5 mM KCl, 1.25 mM NaH_2_PO_4_, 2 mM CaCl_2_, 1 mM MgCl_2_, 25 mM NaHCO_3_, and 11 mM glucose) at 30°C for recovery for at least 1 h before LTP or 2 h before LTD recording. Prior to recording, slices were transferred to a recording chamber where they were perfused continuously with oxygenated ACSF2 at a flow rate of ~3 ml/min at 30°C. Hippocampal CA1 fEPSP was evoked at 0.033 Hz with a 125 μm platinum/iridium concentric bipolar electrode (FHC, Bowdoinham, ME) placed in the middle of stratum radiatum of CA1. Synaptic responses were recorded with ACSF2-filled microelectrodes (1–2 MΩ), positioned ~200 μm away (orthodromic) from the stimulating electrode, and were quantified as the initial slopes of fEPSPs. Input/output relationships were obtained for each slice with various stimulus intensity and responses were set to ~45% max for LTP experiments and ~55% max for LTD experiments. LTP induced by TBS consists of four trains of 10 bursts at 5 Hz, with each burst consisting of four stimuli given at 100 Hz and a 10-s inter-train interval. LTD induced by LFS consists of 900 single pulses at 1 Hz. All plasticity experiments are presented as responses normalized to the average of the 20-min baseline. The 5-min averages taken at the indicated time were used to calculate the magnitude of plasticity and for statistical tests. Paired-pulse responses were recorded with inter-stimulus intervals of 25–250 ms. PPR data were presented as a ratio of the second response slope relative to the first.

### 2.9. Morphology analysis

Coronal sections of 250 μm thickness brain slices were obtained from adult (3 months old) Thy1GFP x *Ica1* mouse brains and mounted on slides preserved with PermaFluor mounting medium (Thermo Scientific). Z series images of GFP expressing CA1 neurons were obtained by a 40 × glycerol objective (512 x 512 in x and y, and a z-step of 0.5 μm) with tile scans to visualize entire dendritic fields using a Zeiss LSM 880 confocal microscope and Zen imaging software (Carl Zeiss). For morphological analysis, 3D reconstructions and quantifications of the dendritic arbors on dendrite length, tip number, and complexity were performed using Imaris 9.5 software (Bitplane). For spine density analysis, high-magnification and high-resolution images (digital zoom 2 at 1,024 × 1,024 in x and y, and a z-step of 0.5 μm) were taken on the first few splits of apical secondary dendrites (around 50–150 μm away from the soma) that are parallel to the imagining plane. All apparent protrusions from dendrites regardless of the shape were counted as spines in the z-series images and normalized to the length of individual dendrite. Dendrite length >200 μm per neuron were quantified. Dendrite and spine analysis were collected from four neurons per animal and from a total of four pairs WT/KO littermates. All reconstructions and spine counts were performed blinded, and tail samples for all animals used were saved for genotype confirmation after the completion of experiments.

### 2.10. Mouse behavioral tests

Behavioral experiments were performed on 2- to 5-month-old *Ica1* WT or KO littermates, group housed at two to five animals per cage containing both genotypes after wean. Except for open-field test, all mice were handled for 3 min each day for 3–4 consecutive days before beginning experiments. All tests were conducted at the animal behavioral facilities of the authors' institutes performed with the examiners blind to animal genotypes. When multiple tests were performed on the same cohort of mice, the tests were performed in the following order: open-field, Y-maze, elevated plus maze, and inhibitory avoidance. All results are collected from two to three cohort of mice.

#### 2.10.1. Open-field

Individual mouse was placed in a photobeam-equipped plastic chamber (45 x 45 cm, PAS open field system, San Diego Instruments) and was allowed to explore free from interference for 30 min. The peripheral area (425 cm^2^) was defined by the two side-photo beams, #1-2 and #15-16, while the central area (1,600 cm^2^) was defined by photo beams #3-14 in each direction. Movements and rearing behavior were tracked using an SDI Photobeam Activity System (San Diego Instruments).

#### 2.10.2. Y-maze

Individual mouse was first placed in an empty holding cage for 2 min before transferring to the start arm of a transparent Y-shaped maze with either left or right arm blocked for free exploration of the two arms for 5 min. All three arms are equal in shape and size with unique visual cues plated outside of each arm. Mouse was then transferred to the holding cage for 2 min before returning to the Y-maze for free exploration of all three arms for 3 min. The time spent and distance traveled in each arm were automatically tracked and analyzed using ANY-maze (Stoelting Co., Wood Dale, IL) tracking software. Discrimination index was calculated as % time in novel arm/(% time in novel arm + % time in familiar arm).

#### 2.10.3. Elevated plus maze

Individual mouse was placed in the center of an elevated plus maze, consists of two closed arms [48 (L) × 10 (W) × 38 (H) cm] and two open arms [48 (L) × 10 (W) cm] and a center platform (10 × 10 cm). After 5-min free exploration, the total time spent and a number of entries into the closed and open arms were automatically recorded and analyzed in ANY-maze.

#### 2.10.4. Inhibitory avoidance

The step-through IA was performed as previously described with minor modifications (Chiu et al., 2017). Briefly, the IA apparatus (GEMINI avoidance system, San Diego Instruments) consisted of a metal grid floor as well as a light chamber and a dark chamber connected by a guillotine-style door. For habituation (day 1), a mouse was placed in the light chamber for free exploration until it crossed to the dark side, which triggered the door to be closed. The latency to crossover was automatically recorded, and the mouse was returned promptly to the home cage. For training (day 2), the mouse was reintroduced to the light chamber and the latency of crossover was recorded. Additionally, the mouse received a 2 s, scrambled, mild 0.4-mA foot shock following the entry of the dark chamber. The mouse remained in the dark chamber for 15 s after shock before returning to the home cage. For testing (day 3), 24 h after training the mouse was reintroduced to the light chamber. The latency of crossover to the dark chamber was recorded as a measure of associative learning and memory performance. The maximum latency was set at 5 min.

### 2.11. Quantification and statistical analysis

All data were presented as mean ± SEM (standard error of the mean). All statistical details and statistical significance, calculated using Mann–Whitney test, unpaired *t*-test, one-way or two-way ANOVA, were indicated in the figure legends. Fisher's LSD and Bonferroni *post-hoc* tests were used following one-way and two-way ANOVA, respectively. ^*^p < 0.05; ^**^p < 0.01, ^***^p < 0.001.

## 3. Results

### 3.1. ICA69 regulates the stability and distribution of PICK1

**Figure 1:**
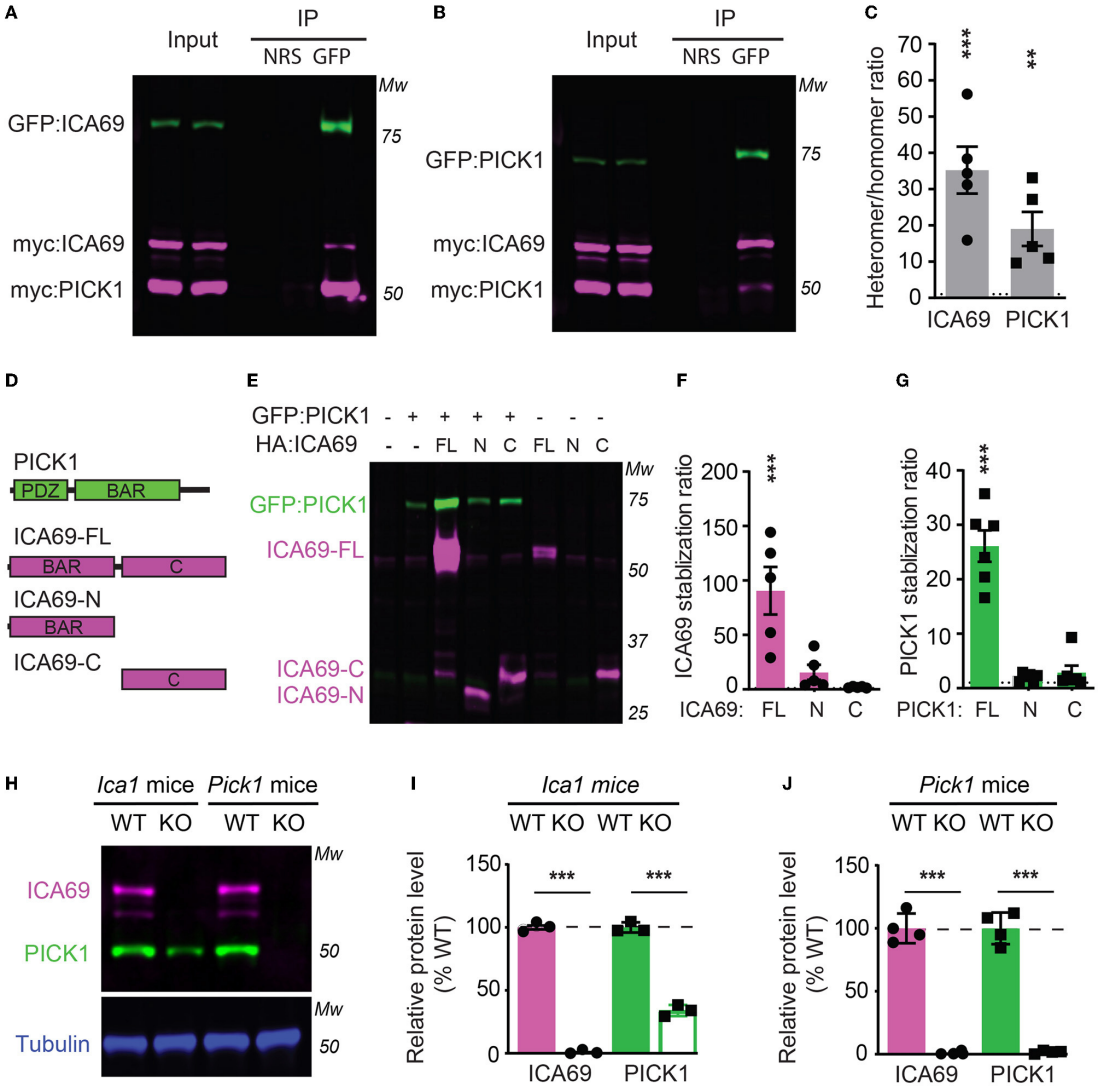
ICA69 and PICK1 heteromerize with and stabilize each other. **(A–C)** Co-immunoprecipitation of HEK cells transfected with either GFP-ICA69, myc-ICA69, and myc-PICK1 **(A)**, or GFP-PICK1, myc-PICK1, and myc-ICA69 **(B)**. Quantifications of heteromer/homomer ratios **(C)** were calculated from input-normalized IP signals. ICA69: 35.2 ± 6.5, *p* < 0.0001; PICK1 = 19.0 ± 4.7, *p* = 0.005, *n* = 5, unpaired *t*-test. NRS represents normal rabbit serum for IP control, and GFP represents GFP antibody for IP GFP-ICA69 or GFP-PICK1. The dashed line indicates ratio = 1. **(D)** Schematics of PICK1 and ICA69 full-length (FL), N-terminus (N), and C-terminus **(C)** protein structures. **(E–G)** ICA69 and PICK1 protein levels in single or co-transfected HEK cells. Stabilization ratios indicate band intensities from co-transfected cells over single-transfected cells. ICA69 stabilizations ratio: ICA69-FL = 90.6 ± 21.8, *p* = 0.0002; ICA69-N = 15.7 ± 6.9, *p* = 0.83; and ICA69-C = 2.0 ± 0.3, *p* > 0.99; PICK1 stabilization ratios: ICA69-FL= 26.1 ± 2.9, *p* < 0.0001; ICA69-N = 2.0 ± 0.3, *p* = 0.97; and ICA69-C = 2.9 ± 1.3, *p* = 0.84; *n* = 5&6/group, one-way ANOVA. The dashed line indicates ratio = 1. **(H–J)** ICA69 and PICK1 protein levels in forebrains of adult *Ica1* or *Pick1* mice. In *Ica1*-KO mice, ICA69 is absent and PICK1 was reduced. % WT: ICA69 = 0.7 ± 0.7, *p* < 0.0001; PICK1 = 34.0 ± 2.3, *p* < 0.0001. In *Pick1*-KO mice, both PICK1 and ICA69 were absent. % WT: ICA69 = 0.8 ± 0.5, *p* < 0.0001; PICK1 = 1.8 ± 0.6, *p* < 0.0001; *N* = 3&4/mouse line, unpaired *t*-test.

To characterize how ICA69 interacts with PICK1, we transfected HEK cells with GFP-tagged ICA69 (GFP-ICA69) as well as myc-tagged ICA69 and PICK1 (myc-ICA69 and myc-PICK1). The GFP tag allows us to perform an immunoprecipitation (IP) experiment of GFP-ICA69 with a GFP antibody and causes a 27 KDa weight shift from its native molecular weight on Western blots. The myc tag allows us to evaluate the relative amount of myc-ICA69 and myc-PICK1 in the GFP pull-down to quantify the amount of ICA69-GFP in homomerization with myc-ICA69 or heteromerization with myc-PICK1. We found that GFP-ICA69 showed approximately 35-fold higher binding to myc-PICK1 than myc-ICA69, indicating that ICA69 has a strong preference for heteromerization with PICK1 rather than homomerization with itself (Figures 1A, C). Likewise, we transfected HEK cells with GFP-PICK1 as well as myc-PICK1 and myc-ICA69. We found that GFP-PICK1 showed a 19-fold higher binding to myc-ICA69 than myc-PICK1, indicating that PICK1 also has a high preference for heteromerization with ICA69 (Figures 1B, C).

To study the structure and function of ICA69, we generated ICA69 truncations containing either the N-terminus (ICA69-N) BAR domain or the C-terminus (ICA69-C) which has no apparent homology to known proteins (Figure 1D). When we first ectopically expressed these constructs alone or with PICK1, we found that the full-length ICA69 (ICA69-FL) protein expression was approximately 90-fold higher when PICK1 was co-transfected than when ICA69-FL was transfected alone (Figures 1E, F). Although not statistically different, ICA69-N also showed a slightly higher expression when co-transfected with PICK1, compared to its barely detectable level when transfected singly (Figures 1E, F). Similarly, we found that the PICK1 protein becomes 27-fold higher when ICA69-FL, but not when ICA69 truncations were co-transfected (Figures 1E, G). As all the constructs are driven by the same promoter, the higher protein level of ICA69 or PICK1 most likely reflects a post-translational regulation rather than transcriptional or translational regulation. Moreover, we found that PICK1 was significantly reduced in the hippocampus of *Ica1*-KO mice and ICA69 is completely absent in the *Pick1* KO mice (Figures 1H–J). These observations in the mouse hippocampus are similar to previous reports in the mouse islet and fly protocerebrum (Cao et al., 2007; Holst et al., 2013). In the fly, normal mRNA transcripts were detected despite the reduced or lost protein levels of PICK1 and ICA69 detected in ICA69-knockdown and PICK1-knockdown flies, respectively (Holst et al., 2013). Because ICA69 and PICK1 have a high tendency to bind to each other, these results suggest that ICA69 and PICK1 heteromerization promotes the protein stability of each other.

**Figure 2:**
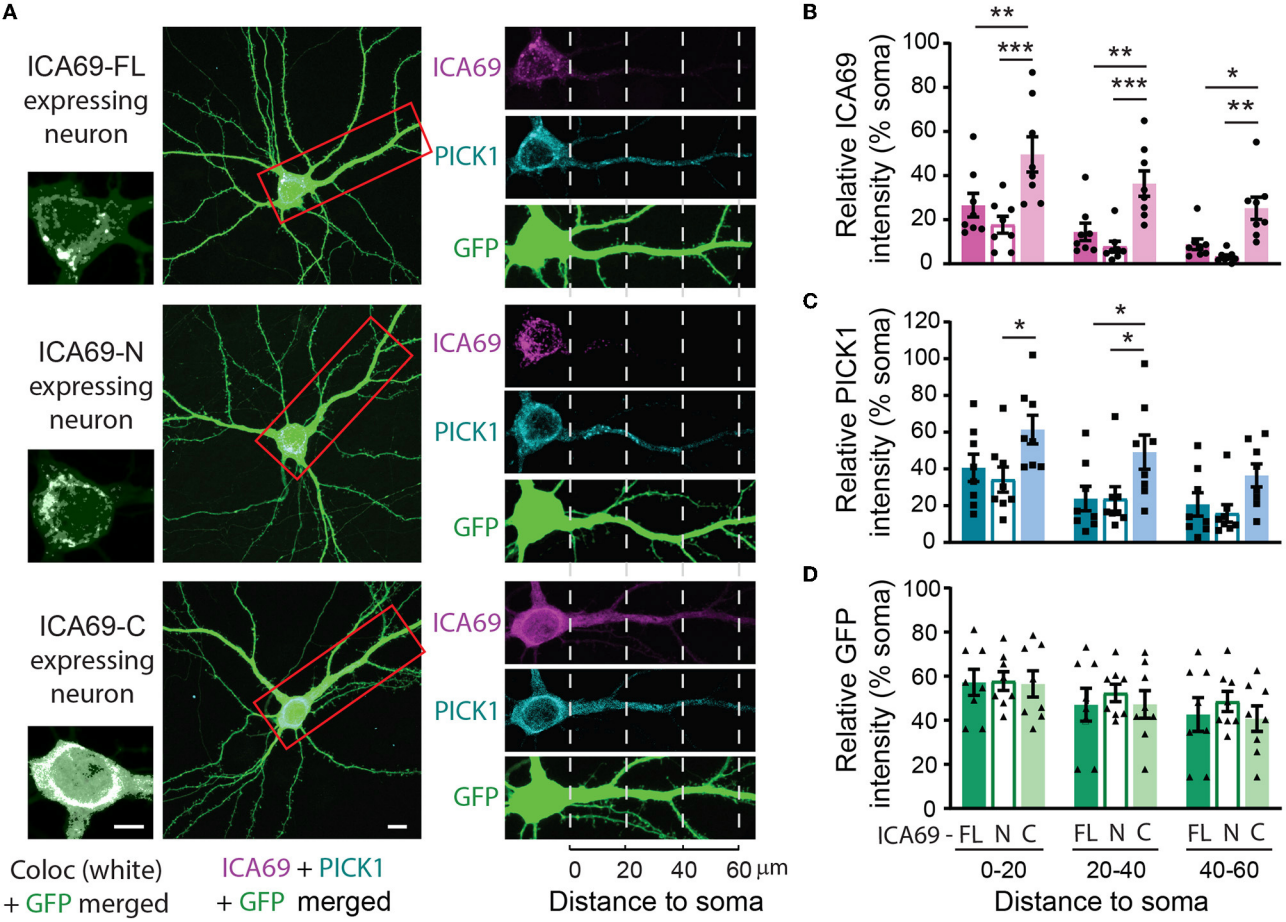
ICA69 localizations affect PICK1's distributions. **(A)** Representative images of hippocampal neurons co-expressing HA-ICA69 (FL, N, or C), myc-PICK1, and cytosolic GFP. Soma areas are magnified on the left to show high levels of colocalization of ICA69 and PICK1 (white). Primary dendrites with soma areas are magnified on the right to show dendritic distributions of ICA69 (magenta), PICK1 (blue), and GFP (green) in separate color channels. Scale bars represent 10 μm in the soma images and 5 μm in the whole neuron images. **(B)** Dendritic distributions of ICA69-FL, -N, or -C quantified as the mean intensities of each dendritic segment relative to intensities in the soma. 0–20: FL = 26.6 ± 5.4 vs. C = 49.7 ± 8.0, *p* = 0.002; *N* = 17.7 ± 3.8 vs. C, *p* < 0.001; 20–40: FL= 14.5 ± 4.0 vs. *C* = 36.4 ± 5.8, *p* = 0.0042; *N* = 7.8 ± 2.5 vs. C, *p* = 0.001; 40–60: FL = 8.9 ± 2.4 vs. C = 25.2 ± 5.0, *p* = 0.04; *N* = 3.2 ± 0.9 vs. C, *p* = 0.004. **(C)** Dendritic distributions of PICK1 in neurons expressing ICA69-FL, -N, or -C. 0–20: FL = 40.6 ± 7.4 vs. *C* = 61.5 ± 7.8, *p* = 0.10; *N* = 34.2 ± 6.8 vs. C, *p* = 0.02; 20–40: FL = 23.9 ± 6.6 vs. *C* = 49.1 ± 9.3, *p* = 0.03; *N* = 23.7 ± 6.7 vs. C, *p* = 0.03; 40–60: FL= 20.7 ± 6.3 vs. *C* = 36.5 ± 6.2, *p* = 0.25; *N* = 15.8 ± 4.7 vs. C, *p* = 0.10. **(D)** Dendritic distributions of GFP in neurons expressing ICA69-FL, -N, or -C. 0–20: FL= 57.2 ± 5.9, *N* = 57.8 ± 4.3, *C* = 56.5 ± 5.9; 20–40: FL= 47.1 ± 7.5, *N* = 52.4 ± 4.0, *C* = 47.2 ± 6.3; 40–60: FL= 42.6 ± 7.7, *N* = 48.5 ± 4.6, *C* = 40.7 ± 5.8; *p* > 0.05 for all comparisons, *n* = 8 neurons/group; two-way ANOVA.

We next expressed ICA69-FL or truncations together with PICK1 in primary hippocampal neurons. We found that while the majority of ICA69-FL or ICA69-N showed a punctate pattern enriched at the peri-nucleus and proximal dendrite, ICA69-C appeared to be much more diffusely distributed throughout the soma and dendrites (Figure 2A). PICK1 is highly co-localized with ICA69, consistent with previous reports (Cao et al., 2007). However, PICK1 had a higher dendritic distribution than all forms of ICA69 (Figure 2A), suggesting that PICK1 may exhibit an ICA69-independent function in dendrites. When the dendritic distribution of ICA69 was quantified using dendritic intensities as a function of distance to soma, the intensities of ICA69-C at all dendritic segments were significantly higher than ICA69-FL and ICA69-N (Figures 2A, B). Interestingly, in ICA69-C expressing neurons, ICA69-C appeared to redistribute PICK1 to more distal dendrites compared with ICA69-FL- or ICA69-N-expressing neurons (Figures 2A, C). Notably, this redistribution effect is specific to PICK1 as we do not see any effect on GFP distributions (Figures 2A, D). Previous yeast two-hybrid and CoIP experiments have reported that either ICA69-N or -C can bind to PICK1 directly (Cao et al., 2007; Wang et al., 2013), our results further demonstrated that ICA69 affects the dendritic distribution of PICK1 and may thus affect its function.

### 3.2. ICA69 is not required for basal synaptic transmission

**Figure 3:**
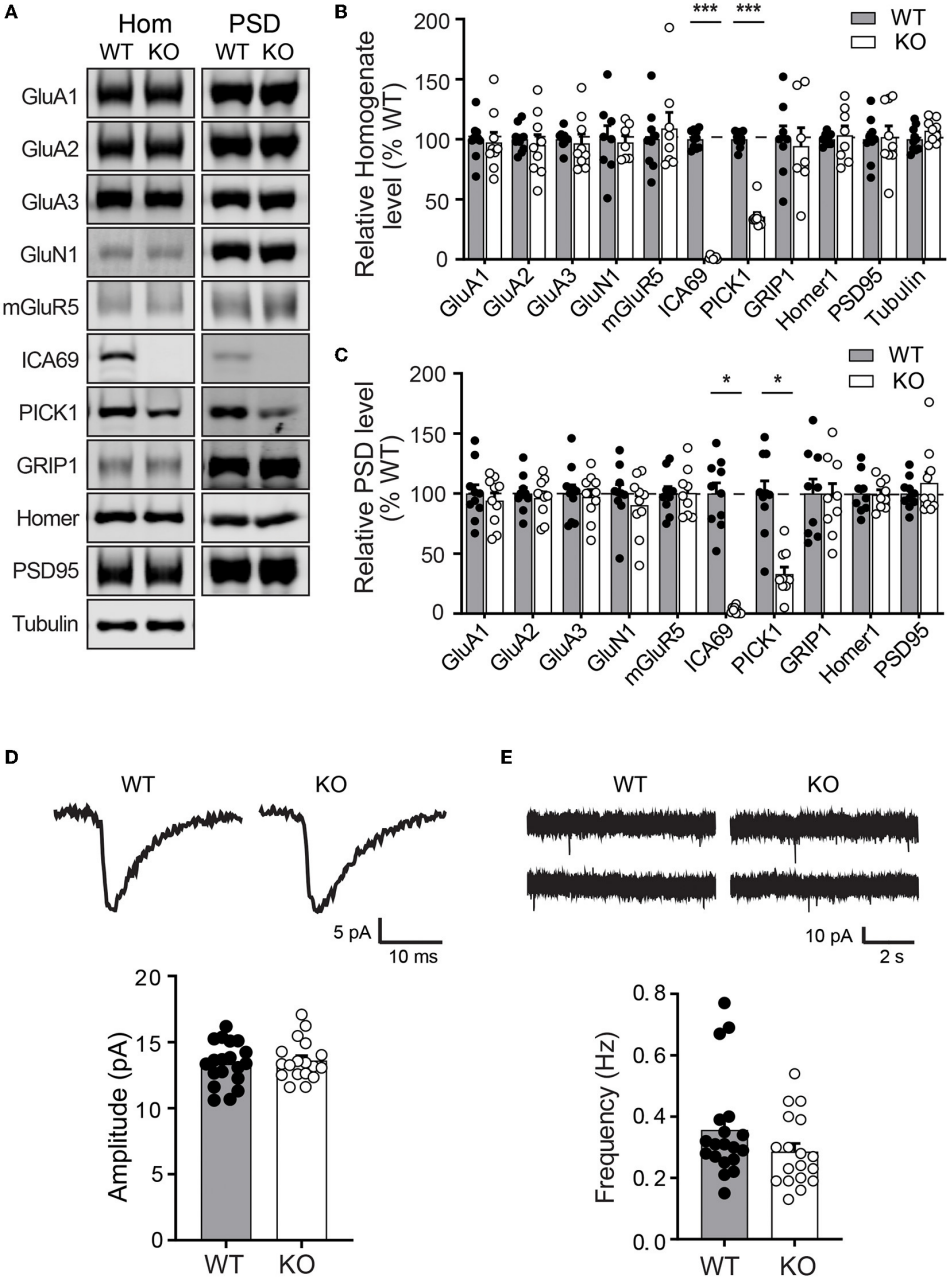
*Ica1-*KO mice have normal basal AMPAR function in the hippocampus. **(A)** Representative Western blots of hippocampal homogenate (Hom) and postsynaptic density (PSD) proteins derived from adult *Ica1* WT and KO littermates. **(B)** Quantifications of Hom proteins in **(A)**. % WT: GluA1 = 97.4 ± 8.3; GluA2 = 95.2 ± 8.7%; GluA3 = 96.7 ± 7.5; GluN1 = 97.4 ± 5.6; mGluR5 = 109.0 ± 13.5; ICA69 = 1.6 ± 0.5, *p* = < 0.001; PICK1 = 35.7 ± 3.3, *p* < 0.001; GRIP1 = 94.3 ± 15.3; Homer1 = 103.3 ± 7.8; PSD95 = 101.9 ± 9.2; *n* = 7–10 mice/group, unpaired *t*-test. **(C)** Quantifications of PSD proteins in **(A)**. % WT: GluA1 = 94.1 ± 6.3; GluA2 = 96.3 ± 5.1%; GluA3 = 97.3 ± 6.1; GluN1 = 90.4 ± 7.8; mGluR5 = 100.4 ± 6.6; ICA69 = 2.2 ± 1.0, *p* = < 0.001; PICK1 = 33.0 ± 5.7, *p* = < 0.001; GRIP1 = 97.6 ± 10.8; Homer1 = 99.1 ± 4.0; PSD95 = 108.8 ± 8.9; *n* = 9–10 mice/group, unpaired *t*-test. **(D)** Representative traces and quantifications of individual mEPSCs recorded from hippocampal CA1 neurons of *Ica1* mice. WT = 13.4 ± 0.4 and KO = 13.6 ± 0.4 pA, *n* = 18–19 neurons from six pairs of WT/KO littermates; *p* = 0.92, Mann–Whitney test. **(E)** Representative traces and quantification of mEPSC frequency. WT = 0.36 ± 0.04 and KO = 0.29 ± 0.03 Hz; *n* = 18–19 neurons from six pairs of WT/KO littermates; *p* = 0.13, Mann–Whitney test.

As ICA69 is a major binding partner of PICK1 and PICK1 is a major scaffold protein for AMPARs, we next examined whether ICA69 regulates the synaptic expression of AMPARs *in vivo*. We isolated hippocampi from *Ica1* wild-type (WT) and KO mice and performed subcellular fractionation assays to compare protein expressions of glutamate receptors and their major scaffolding proteins in total homogenate (Ho) and postsynaptic density (PSD) fractions (Figures 3A–C). As expected, we did not detect ICA69 signal in *Ica1-*KO mice. Consistent with our previous finding that ICA69 is required to stabilize PICK1 in the forebrain homogenate (Figures 1H, I), we found that PICK1 was significantly reduced in both Ho and PSD fractions in the *Ica1*-KO mice (Figures 3A–C). However, we did not find any differences in AMPAR subunits GluA1–3, AMPAR scaffold protein GRIP1, NMDAR subunit GluN1, NMDAR scaffold protein PSD95, metabotropic glutamate receptor 5 (mGluR5), and mGluR5 scaffold protein Homer1 in either the Ho or PSD fraction (Figures 3A–C). These results suggest that ICA69 is not required for targeting or maintaining synaptic levels of glutamate receptors and their scaffold proteins.

To further test whether ICA69 regulates basal AMPAR-mediated synaptic function, we performed whole-cell patch recording on hippocampal CA1 neurons from *Ica1* mice. Spontaneous miniature excitatory postsynaptic currents (mEPSCs) were isolated, and their amplitudes and frequencies were compared between *Ica1* WT and KO littermates. A change in mEPSC amplitude often reflects a change in the number of AMPARs at postsynaptic sites, while a change in mEPSC frequency indicates an alteration in the number of synapses and/or presynaptic vesicle release probability. We found no difference in the mEPSC amplitude between WT and KO mice, suggesting that ICA69 is not required for steady-state postsynaptic AMPAR number (Figure 3D). We observed a trend toward lower mEPSC frequency in the KO mice, but this was not statistically different from the WT mice (Figure 3E). In summary, our biochemical and electrophysiological data show that ICA69 does not regulate synaptic AMPAR composition, number, or function at basal state.

### 3.3. LTP is selectively impaired in *Ica1*-KO mice

**Figure 4:**
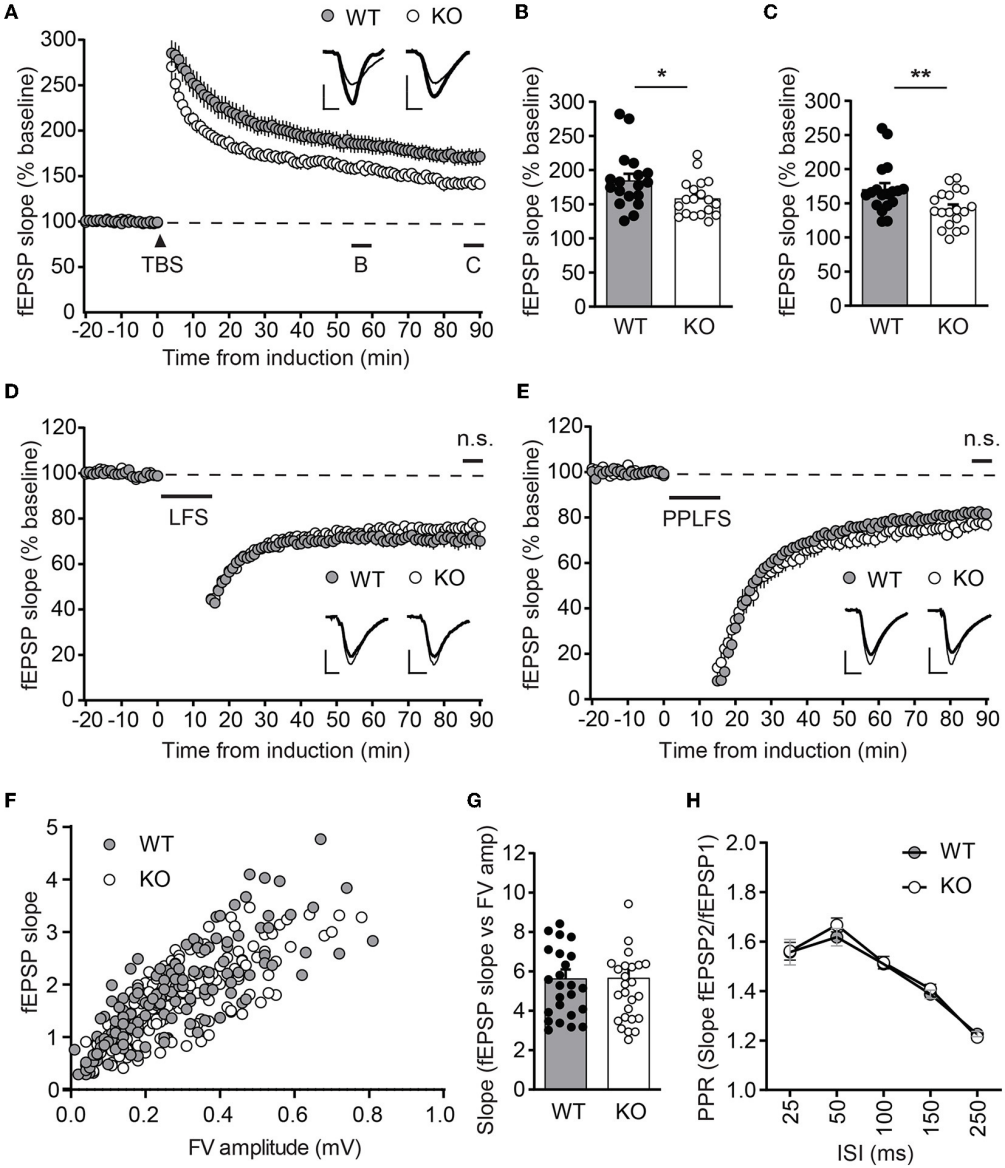
*Ica1*-KO mice exhibit diminished LTP. **(A)** TBS-induced LTP at SC-CA1 synapses from *Ica1* mice. Sample traces represent fEPSPs at one min before (thin) and 90 min after (thick) stimulation. **(B)** The magnitude of TBS-LTP is reduced in *Ica1*-KO mice at 55–60 min. WT = 184.9 ± 9.8 and KO = 158.6 ± 6.2%, *n* = 18–19 slices from seven to eight mice/genotype; *p* = 0.028, unpaired *t*-test. **(C)** The magnitude of TBS-LTP is reduced in *Ica1*-KO mice at 85–90 min. WT = 170.9 ± 8.7 and KO = 142.1 ± 6.0%, *n* = 18–19 slices from seven to eight mice/genotype; *p* = 0.001, unpaired *t*-test. **(D)** LFS-LTD is comparable between *Ica1* WT and KO mice at 85–90 min. WT = 71.0 ± 3.6 and KO = 76.3 ± 1.8%, *n* = 18–21 slices from 10 mice/genotype; *p* = 0.173, unpaired *t*-test. Sample traces represent fEPSPs at 1 min before (thin) and 90 min after (thick) stimulation. **(E)** PPLFS-induced LTD. Sample traces represent fEPSPs at 1 min before (thin) and 90 min after (thick) stimulation. PPLFS-LTD is comparable between *Ica1* WT and KO mice. 85–90 min, WT= 82.0 ± 1.2 and KO = 77.2 ± 2.7%, *n* = 18 slices from seven pairs of WT/KO littermates; *p* = 0.118, unpaired *t*-test. Scale bars represent 0.5 mV (vertical) and 5 ms (horizontal). **(F)** Input–output (I/O) relationships of fEPSPs obtained with different stimulus intensities in *Ica1* WT and KO littermates. **(G)** The slope of the I/O curve is not altered in *Ica1*-KO mice. WT = 5.6 ± 0.5 and KO = 5.7 ± 0.5%, *n* = 25–27 slices from seven to eight mice/genotype; p=0.97, unpaired *t*-test. **(H)** Paired-pulse ratios (PPRs) with different inter-stimulus intervals (ISIs) are comparable between *Ica1* WT and KO littermates. 25 ms: WT = 1.56 ± 0.05 and KO = 1.56 ± 0.04; 50ms: WT = 1.62 ± 0.04 and KO = 1.67 ± 0.03; 100 ms: WT = 1.51 ± 0.02 and KO = 1.52 ± 0.03; 150 ms: WT = 1.39 ± 0.02 and KO = 1.41 ± 0.02; 250 ms: WT = 1.21 ± 0.01 and KO = 1.23 ± 0.02; *n* = 25–27 slices from seven to mice/genotype; *p* > 0.05, two-way ANOVA.

To test whether ICA69 affects activity-dependent AMPAR function, we examined several forms of synaptic plasticity with field recordings at hippocampal Schaffer collateral (SC)-CA1 synapses. We first examined NMDAR-mediated LTP induced by theta burst stimulation (TBS), which is known to require the insertion of postsynaptic AMPARs (Huganir and Nicoll, 2013). Immediately after TBS induction, the field excitatory postsynaptic potential (fEPSP) was similarly potentiated between *Ica1* WT and KO, but the fEPSP was substantially reduced in KO mice for at least 90 min (Figures 4A–C), suggesting that ICA69 is required for proper delivery or maintenance of synaptic AMPARs in response to TBS stimulation. Furthermore, we also examined NMDAR- and mGluR-dependent LTD, which is associated with the endocytosis of AMPARs. However, neither NMDAR-mediated LTD, induced by low-frequency stimulation (LFS), nor mGluR-mediated LTD, induced by paired-pulse low-frequency stimulation (PPLFS) in the presence of APV, was different between KO and WT mice (Figures 4D, E), suggesting a specific role for ICA69 in the activity-dependent incorporation but not the removal of synaptic AMPARs.

Because ICA69 and PICK1 are also expressed at axons, we further tested whether ICA69 regulates presynaptic function by comparing paired-pulse ratios (PPRs), a standard approach to assess neurotransmitter release probability. *Ica1-*KO mice showed comparable PPRs in a wide range of inter-stimulus intervals (Figure 4H). The normal presynaptic properties in *Ica1-*KO mice suggest that ICA69 acts primarily at postsynaptic sites at the SC-CA1 synapses. Additionally, we also examined postsynaptic response vs. presynaptic response in the same hippocampal slice. We do not observe any difference in the input/output relationship (I/O), supporting our previous argument that ICA69 does not regulate basal excitatory synaptic transmission (Figures 4F, G).

### 3.4. Dendrite morphology is normal in *Ica1-*KO mice

**Figure 5:**
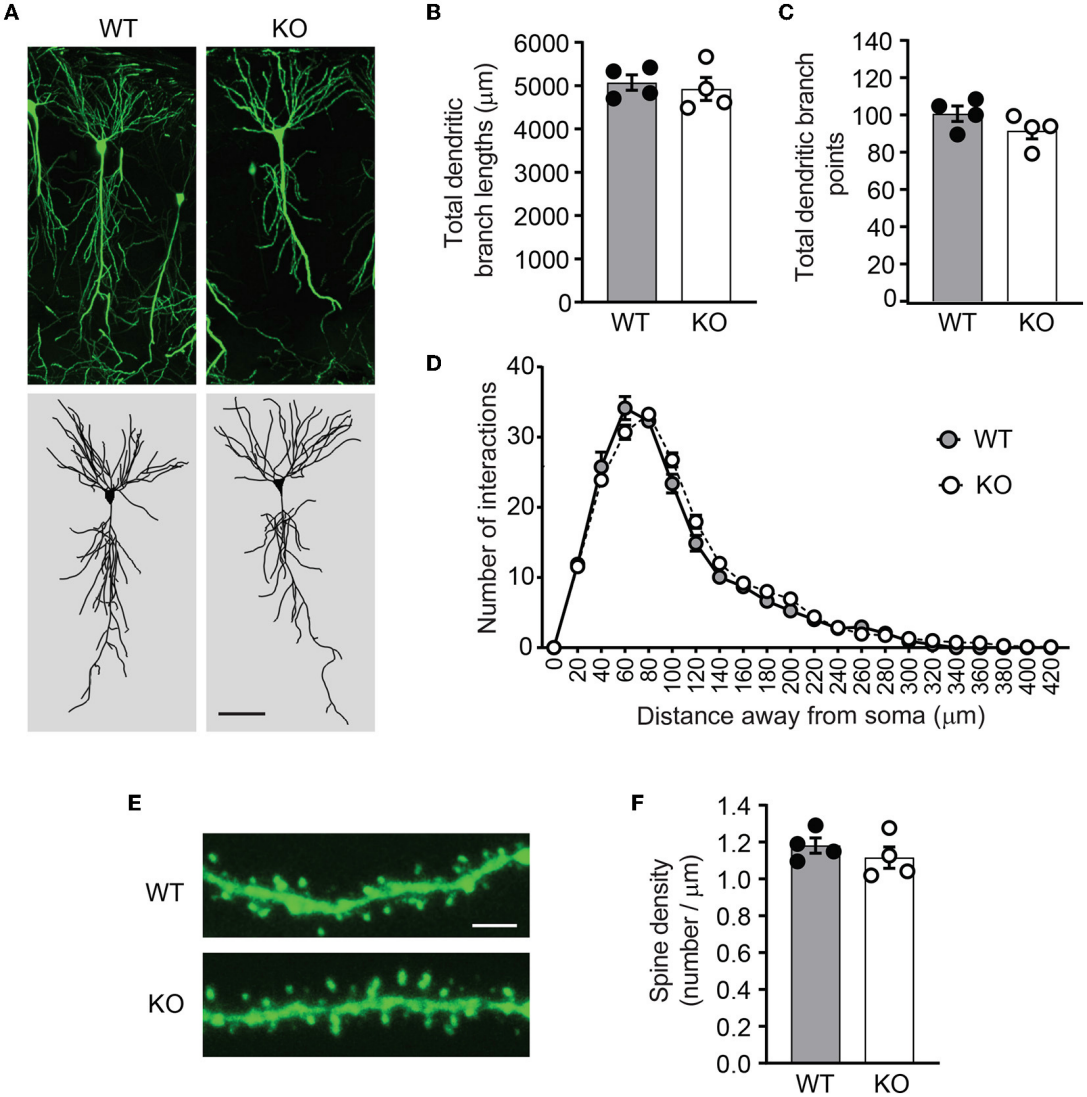
*Ica1-*KO neurons have normal dendrite morphology and spin density. **(A)** Representative images and 3D reconstructions of dendrites of hippocampal CA1 neurons from 3-month-old *Ica1* × Thy1GFP mice. The scale bar represents 50 μm. **(B)** Total dendritic branch lengths of *Ica1* WT and KO neurons are comparable. WT = 5071.8 ± 89.3 and KO = 4924.6 ± 132.0; *n* = 4 neurons per animal from four pairs of WT/KO littermates; p=0.66, unpaired *t*-test. **(C)** Total numbers of dendritic branch points are unaltered in *Ica1*-KO neurons. WT = 100.7 ± 2.1 and KO = 91.6 ± 2.2; *n* = 4 neurons per animal from four pairs of WT/KO littermates; *p* = 0.18, unpaired *t*-test. **(D)** Dendrite branch pattern and complexity quantified by Sholl analyses are unaffected in *Ica1*-KO neurons. *n* = 4 neurons per animal from four pairs of WT/KO littermates; *p* > 0.05, two-way ANOVA. **(E)** Representative images for quantifications of spine densities in *Ica1* × Thy1GFP mice. The scale bar represents 5 μm. **(F)** Spine density is normal in *Ica1*-KO neurons. WT = 1.18 ± 0.02 and KO = 1.12 ± 0.03, *n* = 4 neurons per animal from four pairs of WT/KO littermates; *p* = 0.39, unpaired *t*-test.

PICK1 supports dendrite architecture by inhibiting actin polymerization and filament branching. In cultured neurons, loss of PICK1 significantly alters dendrite morphology and displays more dendritic processes near the soma (Rocca et al., 2008). To test whether ICA69 regulates dendritic morphology *in vivo*, we crossed *Ica1* mice with Thy1GFP-M transgenic mice (Feng et al., 2000) to sparsely label CA1 neurons with GFP (Figure 5A). After imaging and 3D reconstruction of entire dendritic arbors from neurons of *Ica1* WT and KO mice, we found that the overall dendrite morphology, as analyzed by the total dendritic branch length (Figure 5B) and branch points (Figure 5C) were comparable between genotypes. We further performed the Sholl analysis used in the previous publication (Rocca et al., 2008) to examine the 3D dendritic arborization pattern and complexity of CA1 neurons from *Ica1* WT and KO mice, but failed to detect any difference between genotypes (Figure 5D). Finally, we quantified the number of spines on secondary dendrites to measure the density of glutamatergic synapses on CA1 neurons. We also observed no difference between neurons from *Ica1* WT and KO mice (Figure 5E), consistent with our mEPSC frequency results (Figure 3E). Together, these data indicate that ICA69 does not regulate dendrite morphology and glutamatergic synapse number.

### 3.5. Hippocampus-dependent learning and memory significantly impaired in *Ica1*-KO mice

**Figure 6:**
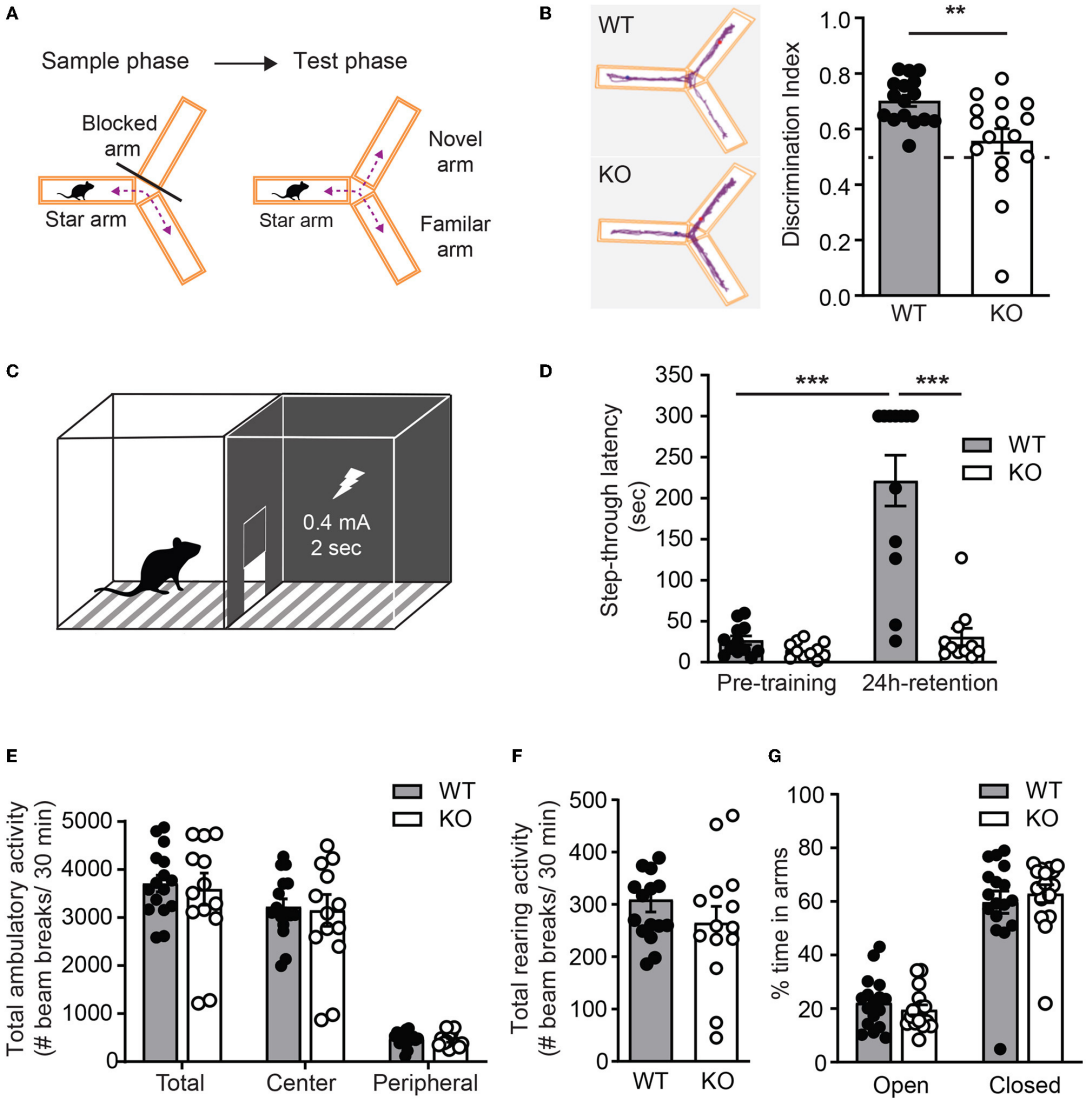
*Ica1*-KO mice have impaired hippocampal-dependent learning and memory. **(A)** Y-maze test protocol. **(B)** Spatial memory, measured as the preference of the animals toward the novel arm, is impaired in *Ica1*-KO mice. Discrimination index: WT = 0.70 ± 0.02 and KO = 0.56 ± 0.04; *p* = 0.006, Unpaired *t*-test. The dashed line indicates chance level (0.5). **(C)** Inhibitory avoidance (IA) setup. **(D)** Associative learning and memory, measured as the tendency of animals to avoid the dark chamber where they were shocked during IA training, are impaired in *Ica1*-KO mice. WT pre-training = 26.7 ± 5.3 vs. WT 24-h retention = 221.4 ± 31.0, *p* < 0.001; KO pre-training = 15.0 ± 2.9 sec vs. KO 24-h retention = 30.9 ± 10.5 sec, *p* = 0.92; WT pre-training vs. KO pre-training, *p* = 0.96; WT 24-h retention vs KO 24-h retention, *p* < 0.001; *n* = 11–12 mice/genotype, two-way ANOVA. **(E)** Total, central, and peripheral ambulatory activities in an open field, measured as the number of horizontal beam breaks, are comparable between *Ica1* WT and KO mice. Total: WT = 3708.6 ± 173.9 and KO = 3595.6 ± 333.2; Center: WT = 3225.6 ± 163.2 and KO = 3150.7 ± 329.9; Peripheral: WT = 482.9 ± 37.6 and KO = 444.9 ± 39.1; *p* > 0.05, two-way ANOVA. **(F)** Exploratory rearing activities in the open-field test, measured as the number of vertical beam breaks, are comparable between *Ica1* WT and KO mice. WT = 309.1 ± 23.6 and KO = 264.7 ± 31.8; *n* = 14–16 mice/genotype; *p* = 0.26, unpaired *t*-test. **(G)** Anxiety-like behavior measured in the elevated plus maze as the % time spent in open arms over closed arms is normal in *Ica1*-KO mice. Open arms: WT = 22.1 ± 2.3 and KO= 19.6 ± 1.9%; closed arms: WT = 59.7 ± 4.2 and KO = 62.9 ± 3.3%, *n* = 16–17 mice/genotype; *p* > 0.05, two-way ANOVA.

To test whether ICA69 regulates cognitive functions *in vivo*, we performed a battery of behavioral experiments to examine hippocampus-dependent cognitive function in *Ica1* WT and KO littermates. A Y-maze consisting three identical arms with distinct visual cues was used to examine their spatial memory. Test mice were first placed at the start arm of the Y-maze with one arm blocked for free exploration of the maze for 5 min during the sample phase. After a 2-min break, the blocker was removed and mice were returned to the maze for free exploration of all arms during the test phase (Figure 6A). *Ica1* WT mice, as expected, showed a high preference of ~0.70 for the novel arm in the test phase due to the natural preference of mice to explore novel environments. *Ica1*-KO mice, however, appeared to have difficulty discriminating between the novel and familiar arms. The discrimination index of 0.56 is just slightly above the 0.5 chance level, demonstrating an apparent impairment in hippocampus-dependent spatial memory (Figure 6B). Furthermore, we used inhibitory avoidance (IA), a single trial associative learning task previously shown to induce LTP and synaptic AMPAR incorporation in the dorsal hippocampus (Whitlock et al., 2006) to test hippocampus-dependent associative learning and memory. Adult mice were first habituated to an arena consisting of light and dark chambers separated by a guillotine-style gate (Figure 6C). When introduced to the light side, both WT and KO littermates quickly entered the dark chamber due to a natural preference for a dark environment. No detectable difference during the pre-training period indicates that *Ica1*-KO mice had comparable motivation and motor function as their WT littermates to enter the dark chambers (Figure 6D). During IA training, mice received a mild foot-shock (0.4 mA, 2s) upon entering the dark chamber. All mice showed vocalization and jumping behavior, suggesting that they had received proper shock training. When tested 24 h after the shock, WT animals displayed a long step-through latency, showing clear and strong associative memory, while *Ica1*-KO mice showed no increase in step-through latency, indicating their lack of IA learning and/or memory (Figure 6D). Although PICK1 is known to regulate chronic pain, acute pain, which can compromise shock sensation, is well preserved in *Pick*1-KO mice (Wang et al., 2011). As ICA69 protein is completely absent in *Pick*1-KO mice (Figure 1J), this suggests that ICA69 is not required for acute pain and further supports our conclusion regarding the role of ICA69 in hippocampus-dependent associative learning and memory. Notably, we found no difference in general locomotion, exploratory activity, and anxiety level in the open field (Figures 6E, F) and elevated plus maze (Figure 6G) tests in *Ica1-*KO animals. Taken together, these results highlight the specific effect of ICA69 on hippocampus-dependent spatial and associative learning and memory.

## 4. Discussion

AMPAR-mediated synaptic function is dynamically regulated as AMPARs traffic in and out of the synaptic membrane under basal conditions or during synaptic plasticity. AMPAR trafficking involves complex pathways that are highly dependent on interactions between the intracellular C-termini of receptors and associated scaffold protein complexes (Diering and Huganir, 2018). PICK1, for example, contains a PDZ domain that binds directly to the PDZ ligand at the C-terminus of GluA2 or GluA3 (Xia et al., 1999). PICK1's BAR domain tends to form a banana-shaped crescent through homo- or heteromerization with itself or other BAR domain proteins to regulate AMPAR-containing endosomes at multiple cellular processes. It has been well established that PICK1 regulates LTD through sensing/binding curved membranes, retaining intracellular AMPARs, and inhibiting AMPAR recycling (Steinberg et al., 2006; Citri et al., 2010; Volk et al., 2010; Anggono et al., 2013). However, the underlying mechanisms by which PICK1 regulates LTP remain largely unexplored. Given ICA69's function in regulating the forward secretory trafficking of insulin- and growth hormone-containing vesicles together with PICK1 in the pancreas and pituitary, ICA69 likely plays a role in the forward secretory trafficking of AMPARs to support the surface or synaptic delivery of AMPARs during LTP. Utilizing multidisciplinary *in vivo* and *in vitro* approaches, we found that ICA69 regulates the subcellular distribution and stability of PICK1. In contrast to PICK1's bidirectional regulation of synaptic plasticity (Terashima et al., 2008; Volk et al., 2010), ICA69, in collaboration with PICK1, selectively regulates LTP and has a significant impact on hippocampus-dependent learning and memory.

Basal excitatory synaptic transmission is largely regulated by constitutive AMPAR trafficking pathways (Diering and Huganir, 2018). We observed comparable levels of major AMPAR subunits in the PSD as well as comparable amplitudes of AMPAR-mEPSCs in *Ica1* WT and KO mice from our biochemical and electrophysiological experiments. These results indicate that ICA69-mediated AMPAR trafficking is independent of constitutive AMPAR trafficking in the hippocampus. Consistently, genetic deletion of PICK1 or molecular knockdown of PICK1 in juvenile or adult rodents also showed normal basal synaptic transmission in the hippocampus (Citri et al., 2010; Volk et al., 2010). These results demonstrate that ICA69 and PICK1 are not required to support or maintain AMPAR number at excitatory synapses under a resting state in the hippocampus. Although PICK1 was reported to facilitate basal surface expression of AMPARs by promoting GluA2 maturation and ER exist in cultured hippocampal neurons (Lu et al., 2014), this regulation does not seem to play a dominant role in rodent hippocampus at the resting state. Notably, ICA69 or PICK1 overexpression in primary cultured neurons reduces the surface, but not total expression of GluA2-containing AMPARs (Cao et al., 2007). Thus, increased ICA69 or PICK1 may retain GluA2-containing AMPARs intracellularly and alter the composition or number of synaptic AMPARs in the culture system. Further experiments are needed to investigate the physiological implications of overproduced ICA69 or PICK1 in shifting AMPAR composition or influencing basal synaptic transmission *in vivo*.

Synaptic plasticity at hippocampal SC-CA1 synapses is mainly regulated by activity-dependent insertion or removal of AMPARs (Huganir and Nicoll, 2013; Diering and Huganir, 2018). We have examined NMDAR-dependent LTP and LTD as well as mGluR-dependent LTD in adult *Ica1* mice and found a selective role for ICA69 in NMDAR-dependent LTP but not in either form of LTD. These results are somewhat surprising given the well-defined role of PICK1 in endocytosis and its contribution to both the NMDAR and mGluR-LTD in various brain areas (Steinberg et al., 2006; Citri et al., 2010; Volk et al., 2010; Anggono et al., 2013). Another differential role of PICK1 and ICA69 is in neuronal structure, in which PICK1, but not ICA69, is required for dendrite morphology. Notably, PICK1 proteins are lost or significantly reduced in *Ica1*-KO mice and ICA69-knockdown flies (Cao et al., 2013; Holst et al., 2013). These results suggest that the remaining amount of PICK1 in *Ica1* KO or ICA69 knockdown may be sufficient to remove AMPARs for proper expression of NMDAR- and mGluR-LTD, as well as to inhibit actin polymerization for proper dendrite architecture without the need of ICA69. Moreover, although PICK1 shows a high degree of colocalization with ICA69 in the Golgi and Golgi-derived vesicles in the soma and dendritic shafts, PICK1 appears to have a distinct population localized to spines (Cao et al., 2007) and distal dendrites (Figure 2). Thus, there may be at least two distinct pools of PICK1/AMPAR protein complexes. The pool that interacts with ICA69 is restricted and stored in the Golgi or Golgi-derived vesicles to be used to supply AMPARs during LTP. The early reduction in the LTP magnitude observed in *Ica1-*KO mice (Figure 4A) suggests that the ICA69-regulated AMPAR pool likely represents a more readily mobilized pool in NMDAR-dependent synaptic strengthening in the hippocampus. It will be interesting to test whether protein synthesis-dependent LTP is even more severely impaired, as the newly synthesized AMPARs are expected to be affected in addition to the readily mobilized AMPAR pool in *Ica1*-KO mice. On the other hand, the other pool that does not interact with ICA69 is located at or near synapses and could therefore be involved in synaptic AMPAR removal during NMDAR- or mGluR-dependent LTD at the hippocampal SC-CA1 synapses. Notably, ICA69-FL or ICA69-C fusion proteins impair cerebellar LTD at the inhibitory Purkinje neuron synapses (Wang et al., 2013), suggesting that ICA69 may play different roles in synaptic plasticity in a brain area and/or cell type-specific manner.

What is the molecular mechanism underlying ICA69-regulated LTP in the hippocampus? One possibility is that ICA69 may facilitate the forward trafficking of PICK1/AMPAR-containing endosome formation or trafficking toward cell membrane to support synaptic AMPAR delivery during LTP, similar to its role in promoting the secretory trafficking of insulin- or growth hormone-containing vesicles from ER, Golgi to post-Golgi in the pancreas and pituitary (Cao et al., 2013; Holst et al., 2013). Alternatively, but not mutually exclusive, ICA69 may serve as a negative regulator to retain and store PICK1/AMPAR protein complexes in Golgi or Golgi-derived vesicles. Upon LTP stimulation, ICA69 releases PICK1/AMPAR complexes to allow AMPARs to traffic out of the reserved intracellular pool for subsequent AMPAR insertion. In cultured neurons, AMPARs reportedly enter the dendritic secretory pathway and accumulate in recycling endosomes following ER exit (Bowen et al., 2017). Interestingly, glycine-induced LTP moves PICK1 into Rab11-containing recycling endosomes and the PSD (Jaafari et al., 2012), suggesting that ICA69 and PICK1 may participate in the dendritic secretory machinery to regulate local synaptic AMPAR delivery through recycling endosomes during LTP.

Learning induces LTP in the hippocampus *in vivo* by incorporating more AMPARs into synapses, whereas blocking synaptic delivery of AMPARs is known to impair hippocampus-dependent learning in mice (Whitlock et al., 2006; Mitsushima et al., 2011). *Ica1*-KO mice exhibit significant impairments in two hippocampus-dependent behavior tests, the Y-maze for spatial memory and inhibitory avoidance for associative learning and memory assessment. These results provide direct evidence for the regulation of ICA69 in hippocampus-dependent learning and memory, likely due to the requirement of ICA69 in NMDAR-dependent LTP (Figure 4A). Recently, several human genetics studies have discovered that a *de novo Ica1* mutation and copy number variations are associated with autism spectrum disorder and intellectual disability (Salyakina et al., 2011; Gai et al., 2012; Iossifov et al., 2012; Girirajan et al., 2013; Nava et al., 2014). Therefore, it should be noted that although our conclusions are mostly based on experiments performed in the hippocampus, ICA69 and PICK1 are widely expressed in the central nervous system and may play different roles in different brain areas to influence different behavioral domains. Additionally, loss of ICA69 or PICK1 in mice is known to develop late-onset diabetes (Cao et al., 2013), and elderly diabetics are at a high risk for dementia including Alzheimer's disease (Biessels and Despa, 2018). How might ICA69 and PICK1 regulate complex neurological disorders that involve variable behavioral phenotypes in autism spectrum disorders, intellectual disability, schizophrenia, and perhaps also Alzheimer's disease? Further experiments are needed to characterize ICA69/PICK1's roles in different brain circuits and ages and to investigate their corresponding behavioral correlates, to gain insight into their potential impacts on different forms of synaptic plasticity and brain function in health and disease.

## Data availability statement

The original contributions presented in the study are included in the article/supplementary material, further inquiries can be directed to the corresponding authors.

## Ethics statement

The animal study was reviewed and approved by the Academia Sinica and Johns Hopkins School of Medicine.

## Author contributions

S-LC, C-MC, and RH designed research and wrote the manuscript. S-LC and C-MC performed the research and analyzed the data. All authors contributed to the article and approved the submitted version.
